# Romidepsin targets multiple survival signaling pathways in malignant T cells

**DOI:** 10.1038/bcj.2015.83

**Published:** 2015-10-16

**Authors:** B C Valdez, J E Brammer, Y Li, D Murray, Y Liu, C Hosing, Y Nieto, R E Champlin, B S Andersson

**Affiliations:** 1 Department of Stem Cell Transplantation and Cellular Therapy, University of Texas MD Anderson Cancer Center, Houston, TX, USA; 2 Department of Experimental Oncology, Cross Cancer Institute, Edmonton, Alberta, Canada

## Abstract

Romidepsin is a cyclic molecule that inhibits histone deacetylases. It is Food and Drug Administration-approved for treatment of cutaneous and peripheral T-cell lymphoma, but its precise mechanism of action against malignant T cells is unknown. To better understand the biological effects of romidepsin in these cells, we exposed PEER and SUPT1 T-cell lines, and a primary sample from T-cell lymphoma patient (Patient J) to romidepsin. We then examined the consequences in some key oncogenic signaling pathways. Romidepsin displayed IC_50_ values of 10.8, 7.9 and 7.0 nm in PEER, SUPT1 and Patient J cells, respectively. Strong inhibition of histone deacetylases and demethylases, increased production of reactive oxygen species and decreased mitochondrial membrane potential were observed, which may contribute to the observed DNA-damage response and apoptosis. The stress-activated protein kinase/c-Jun N-terminal kinase signaling pathway and unfolded protein response in the endoplasmic reticulum were activated, whereas the phosphatidylinositol 3-kinase/AKT/mammalian target of rapamycin (PI3K/AKT/mTOR) and β-catenin pro-survival pathways were inhibited. The decreased level of β-catenin correlated with the upregulation of its inhibitor SFRP1 through romidepsin-mediated hypomethylation of its gene promoter. Our results provide new insights into how romidepsin invokes malignant T-cell killing, show evidence of its associated DNA hypomethylating activity and offer a rationale for the development of romidepsin-containing combination therapies.

## Introduction

Romidepsin (FK228 or FR901228) is a depsipeptide small molecule (MW=540.7) that belongs to bicyclic peptide selective inhibitors of Class I histone deacetylases (HDAC). It was originally isolated from *Chromobacterium violaceum* in Japan and later found to exhibit antitumor activity *in vitro* and *in vivo*.^1^ Although its cytotoxicity is not limited to hematological malignancies, it was approved by the US Food and Drug Administration for the treatment of relapsed/refractory cutaneous T-cell lymphoma in 2009 and relapsed/refractory peripheral T-cell lymphoma (PTCL) in 2011. However, the efficacy of romidepsin in other malignant T-cell lines, such as lymphoblastic leukemia/lymphoma, is unknown.

The cytotoxicity of romidepsin is mediated through multiple biological effects invoked by various mechanisms. The disulfide bond of the prodrug romidepsin is reduced inside the cell and generates a thiol functional moiety that reversibly interacts with the zinc atom in the binding pocket of Zn-dependent histone deacetylase, resulting in inhibition of its enzymatic activity.^1, 2^ This inhibition of HDAC may restore normal gene expression in cancer cells and result in cell cycle arrest and apoptosis. Romidepsin induces cell cycle arrest in lung carcinoma cells by increasing the level of p21^Waf1/Cip1^ and hypophosphorylated Rb.^3^ Apoptosis is induced through the production of reactive oxygen species (ROS) in HL-60 leukemia cells^4^ and urinary bladder cancer cells,^5^ which concomitantly cause mitochondrial membrane dysfunction and caspase activation.^6^

Another mechanism of action for the cytotoxicity of romidepsin is the inhibition of the PI3K/AKT pathway seen in lung and colorectal cancer cells.^7, 8^ Moreover, expression of the pro-survival nuclear factor-kappa B pathway genes was downregulated in cells isolated from cutaneous or peripheral T-cell lymphoma patients treated with romidepsin.^9^

In spite of these proven biological activities of romidepsin, studies on its mechanisms of cytotoxicity are more limited compared with other HDAC inhibitors. Understanding the precise mechanism underlying its activity against malignant T cells may lead to better development of optimized treatment schedules and stimulate the design of more efficacious combination with other cytotoxic agents. Hence, we decided to determine the effects of romidepsin on various survival signaling pathways in malignant T-cell lines and a primary sample from a patient with T-cell lymphoma to generate new insights regarding the mechanism of cytotoxicity of romidepsin in malignant T cells. Our results also provide a basis for the rational development of combination treatments using romidepsin together with other cytotoxic drugs.

## Materials and methods

### Cell lines, patient cell sample and drug

PEER (from Dr Guillermo Garcia-Manero's laboratory, University of Texas MD Anderson Cancer Center) and SUPT1 (ATCC, Manassas, VA, USA) are established cell lines originally isolated from patients with T-cell acute lymphoblastic leukemia and T-cell lymphoblastic lymphoma, respectively.^10, 11^ The PEER cell line is p53-negative and SUPT1 is p53-positive. Primary cell sample (Patient J) was isolated from the peripheral blood of a patient at University of Texas MD Anderson Cancer Center with relapsed Sezary syndrome, an aggressive variant of cutaneous T-cell lymphoma. Patient J received multiple lines of chemotherapy, radiation and allogeneic stem cell transplantation. His circulating Sezary cells were isolated during an aggressive stage and ultimately fatal relapse after his transplantation. All cells were grown in Roswell Park Memorial Institute medium 1640 (Mediatech, Manassas, VA, USA) supplemented with 10% heat-inactivated fetal bovine serum (Sigma-Aldrich, St Louis, MO, USA) and 100 U/ml penicillin and 100 μg/ml streptomycin (Mediatech) at 37 °C in a fully humidified atmosphere of 5% CO_2_ in air.

Mononuclear cells were purified from a patient-derived cell sample using lymphocyte separation medium (Mediatech) and incubated in suspension in Roswell Park Memorial Institute medium 1640 medium as described above. The sample was obtained after obtaining written informed consent, and all studies using this patient sample were performed under a protocol approved by the Institutional Review Board of the University of Texas MD Anderson Cancer Center, in accordance with the Declaration of Helsinki.

Romidepsin was obtained as a 10 mm solution in dimethyl sulfoxide from Selleck Chemicals (Houston, TX, USA), diluted to 1 mm with dimethyl sulfoxide and stored at −20 °C. It was further diluted in culture medium immediately prior to each experiment.

### Cell proliferation and cell death assays

Cell proliferation was determined as previously described using the 3-(4,5-dimethylthiazol-2-yl)-2,5-diphenyl tetrazolium bromide assay.^12^ The inhibition of cell proliferation after 48-h romidepsin exposure was determined relative to the control cells exposed to solvent alone. Its IC_50_ value (the concentration of romidepsin that inhibited 50% proliferation) was calculated using the CalcuSyn software (Biosoft, Ferguson, MO, USA). Cell death was determined by flow cytometric measurements of phosphatidylserine externalization with Annexin-V-FLUOS (Roche Diagnostics, Indianapolis, IN, USA) and 7-aminoactinomycin D (BD Biosciences, San Jose, CA, USA) using a Muse Cell Analyzer (EMD Millipore, Billerica, MA, USA).

### Western blot analysis

Cells were exposed continuously to romidepsin for 48 h, harvested and washed with cold phosphate-buffered saline. Cells were lysed with lysis buffer (Cell Signaling Technology, Danvers, MA, USA). The protein concentrations of cell extracts were determined using a BCA Protein Assay kit (ThermoFisher Scientific, Rockford, IL, USA). Western blot analyses were done as previously described.^12^ The sources of the antibodies and their optimum dilutions are provided in the Supplementary Materials.

### Analysis of ROS

Cells were exposed to various concentrations of romidepsin for 24 h and analyzed for early production of ROS using CM-H2DCFDA (5-(and-6)-chloromethyl-2',7'-dichlorodihydrofluorescein diacetate, acetyl ester), an ROS indicator that diffuses into cells where it is oxidized to a fluorescent product (Life Technologies, Grand Island, NY, USA). In brief, cells were aliquoted (0.5 ml) into 5 ml tubes and 1 μl of 1.5 mm CM-H2DCFDA (dissolved in dimethyl sulfoxide) was added. Cells were incubated at 37 °C for 1 h and immediately analyzed with a Gallios Flow Cytometer (Beckman Coulter, Brea, CA, USA) using excitation/emission wavelengths of 492/520 nm. Geometric means of the fluorescence intensities were compared, and the relative fold increase in ROS production was calculated.

### Analysis of mitochondrial membrane potential (MMP)

Cells were exposed to romidepsin for 48 h, aliquoted (0.5 ml) into 5 ml tubes and then combined with 40 μl of 1:10 diluted MMP-sensitive fluorescent dye JC-1 reagent (5,5′,6,6′-tetrachloro-1,1′,3,3′-tetraethylbenzimidazolylcarbocyanine iodide, Cayman Chemical, Ann Arbor, MI, USA). All samples were incubated at 37 °C for 20 min and immediately analyzed by flow cytometry as described by the manufacturer. As a positive control, 1 μm valinomycin was added to untreated cells and analyzed with JC-1 reagent similarly to the other samples.

### Real-time PCR

Real-time PCR was used to determine the level of expression of *SFRP1* and the methylation status of its gene promoter. For gene expression analysis, total RNA was extracted from cells exposed to romidepsin for 48 h, purified and used for complementary DNA synthesis and RT-PCR as described.^13^ For demethylation analysis, genomic DNA was extracted from similarly drug-exposed cells and analyzed as described.^13^ Primers were as previously described.^13^

### Statistical analysis

Results are presented as the mean±s.d. of at least three independent experiments and statistical analysis was performed using a Student's paired *t*-test with a two-tailed distribution.

## Results

### Antiproliferative/apoptotic efficacy of romidepsin in malignant T cells

**Figure 1:**
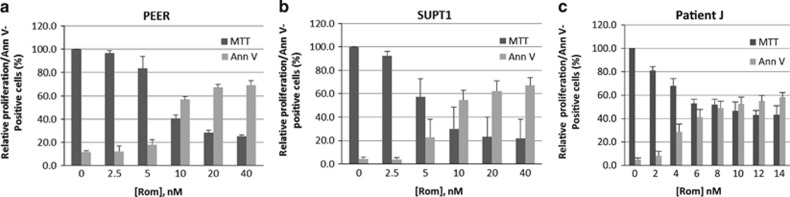
Cytotoxicity of romidepsin in two malignant T-cell lines and one primary T-cell lymphoma patient sample. Cells were exposed continuously to the indicated concentrations of romidepsin (Rom) for 48 h and analyzed for cell proliferation and apoptosis by the MTT and Annexin V (Ann V) assays, respectively. Established cell lines are shown in **a** and **b**, and a cell sample from a T-cell lymphoma patient is shown in **c**.

To determine the cytotoxicity of romidepsin in malignant T cells, we exposed PEER and SUPT1 cells to various drug concentrations for 48 h. Inhibition of cell proliferation was measured by the 3-(4,5-dimethylthiazol-2-yl)-2,5-diphenyl tetrazolium bromide assay and the extent of cell death through apoptosis was analyzed by flow cytometry using Annexin V assay. The two cell lines exhibited similar, remarkably high, sensitivity to romidepsin (Figures 1a and b) with calculated IC_50_ values of 10.8 and 7.9 nm for PEER and SUPT1 cells, respectively. At 10 nm romidepsin, ~57% of PEER cells and ~54% of SUPT1 cells were labeled with Annexin V, suggesting significant apoptosis. The potential clinical relevance of these results is suggested by the cytotoxicity of romidepsin in a primary cell sample of Sezary cells isolated from a patient with cutaneous T-cell lymphoma. An IC_50_ of 7 nm romidepsin was observed in this patient-derived sample, with ~50% of the cells being Annexin V-positive at 10 nm drug (Figure 1c). These results suggest similar and profound sensitivity of both established T-cell lines and patient T-cell sample to low concentrations of romidepsin.

### Effects of romidepsin on protein acetylation and related enzymes

**Figure 2:**
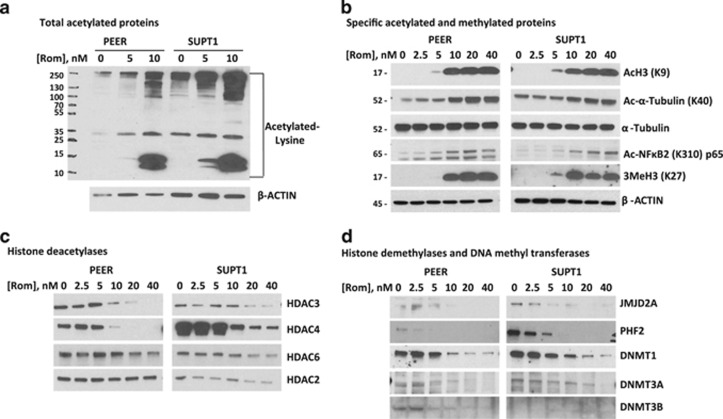
Effects of romidepsin (Rom) on the levels of acetylated proteins and related enzymes. Cells were exposed to romidepsin for 48 h and total cell extracts were analyzed by western blot. The numbers on the left (**a** and **b**) refer to protein molecular weight in kilodaltons. The same cell extracts were analyzed in (**b**, **c** and **d**), and the β-actin loading control is shown in panel **b**.

As romidepsin is a histone deacetylase inhibitor, we explored its effects on the level of acetylated histone and non-histone proteins. Western blot analysis of total cell extracts from PEER and SUPT1 cells exposed to the drug, using an antibody that recognized proteins with acetylated-lysine, showed a dose-dependent increase in the level of multiple acetylated proteins with molecular weights ranging from 10 to 250 kDa (Figure 2a). At 10 nm romidepsin, significant acetylation of proteins with MW=12–15 kDa and 100–250 kDa was observed. To identify some of these proteins, we used antibodies that recognized specific acetylated proteins. Acetylation of histone 3 at lysine 9 occurred in PEER and SUPT1 cells exposed to 5 nm romidepsin, which markedly increased at 10 nm (Figure 2b). Although acetylation of α-tubulin at lysine 40 was observed in untreated control cells, the level of acetylation further increased at 10 nm romidepsin. Increased acetylation of nuclear factor-kappa B2 p65 at lysine 310 was also observed in both cell lines at 10 nm romidepsin and above (Figure 2b).

Acetyl transferases/deacetylases and methyl transferases/demethylases are known to functionally interact.^14^ We therefore sought to determine how acetylation of histone 3 might affect its methylation status. Western blot analysis showed a significant increase in its trimethylation at lysine 27 after romidepsin exposure, which correlates with its acetylation at lysine 9 (Figure 2b). These results suggest that romidepsin influences the acetylation and methylation status of proteins in PEER and SUPT1 cells (Figures 2a and b) and in the T-cell lymphoma patient sample (data not shown).

We next examined possible enzymes that may be involved in the observed increase in protein acetylation and methylation. The level of HDAC3, HDAC4 and HDAC6 decreased in PEER and SUPT1 cells exposed to at least 10 nm romidepsin with minimal effect on the level of HDAC2 (Figure 2c), suggesting some isoform specificity. HDAC3 and HDAC6 are known to be responsible for deacetylation of Ac-nuclear factor-kappa B2 p65 and Ac-α-tubulin^15, 16^ and the observed decrease in their expression correlates with increased levels of their acetylated substrates (Figure 2b). The level of histone demethylases JMJD2A and PHF2 also decreased in cells exposed to the same drug concentrations (Figure 2d), consistent with the observed increase in the level of methylated histone 3 (Figure 2b). DNA methyl transferases DNMT1, DNMT3A and DNMT3B were also downregulated by romidepsin (Figure 2d), suggesting possible effects of romidepsin on the methylation-dependent expression of tumor suppressor genes (see additional results below). Overall, these results show broad epigenetic effects of romidepsin in malignant T cells, which involve protein acetylation and methylation as well as DNA hypomethylation.

### Romidepsin induces production of ROS and decreases MMP

**Figure 3:**
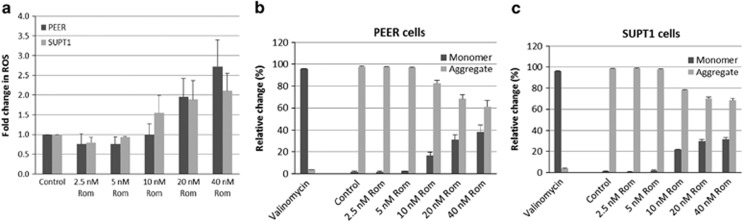
Flow cytometric analysis of cells exposed to romidepsin. Cells were exposed to romidepsin (Rom) for 24 h (**a**) or 48 h (**b** and **c**) and analyzed by flow cytometry for production of reactive oxygen species (ROS) (**a**) and changes in mitochondrial membrane potential (**b** and **c**).

Some histone deacetylase inhibitors are known to cause production of ROS.^17^ To determine whether ROS production might mediate romidepsin-induced cytotoxicity in malignant T-cell lines (Figure 1), cells were exposed to the drug for 24 h, stained with an ROS indicator CM-H2DCFDA, and analyzed by flow cytometry. Figure 3a shows a dose-dependent increase in the level of ROS in PEER and SUPT1 cells starting at 10 nm romidepsin. At 40 nm romidepsin, an approximately twofold increase in ROS production was observed.

This increase in the level of ROS may damage the mitochondrial membrane. We therefore determined whether romidepsin would affect MMP. As we hypothesized that any change in the MMP would be manifested at a later time point, we exposed cells to the drug for 48 h prior to flow cytometric analysis using the JC-1 reagent (see Materials and methods). JC-1 forms aggregates in the mitochondria and dissociates to a corresponding monomeric form in the cytoplasm. Valinomycin, a known ionophore, was used as a positive control; exposure of cells to 1 μm valinomycin for 1 h prior to addition of JC-1 reagent resulted in ~93% monomeric JC-1, suggesting decreased MMP and leakage of the mitochondrial membrane. Relative to the negative control, the abundance of monomeric JC-1 increased starting at 10 nm romidepsin, suggesting decreased MMP (Figures 3b and c). Coincidentally, at this concentration of romidepsin, increased acetylation and methylation of histone 3, increased acetylation of α-tubulin and nuclear factor-kappa B2, and decreased levels of HDAC and demethylases were observed (Figure 2).

### Romidepsin activates the DNA-damage response and apoptosis pathways

**Figure 4:**
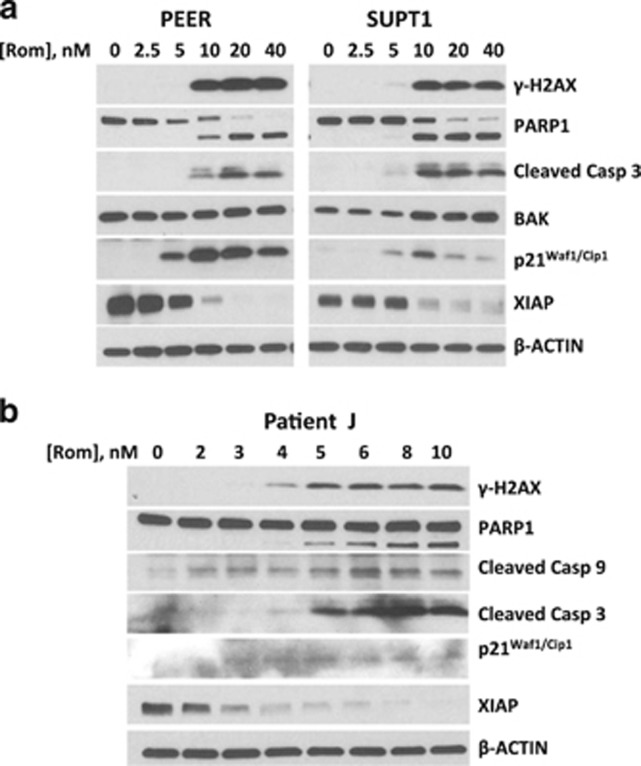
Western blot analysis of proteins involved in DNA-damage response and apoptosis. Established cell lines (**a**) and patient J cell sample (**b**) were exposed to different concentrations of romidepsin (Rom) for 48 h and total cell extracts were analyzed.

As the HDAC inhibitors trichostatin A and sodium butyrate have been shown to induce DNA damage in leukemia cell lines^18^ and the resulting DNA double-strand breaks might be due to slowing down of replication forks,^19^ we sought to determine whether romidepsin would induce DNA damage in malignant T cells. We also hypothesized that the production of ROS (Figure 3a) would augment DNA-damage response activation. Increase in the phosphorylation of histone 2AX (γ-H2AX) is a commonly used indicator of DNA-damage response to double-strand breaks. We therefore examined changes in the level of this protein marker. Exposure of PEER and SUPT1 cells to 10 nm romidepsin (or greater) resulted in a marked increase in the level of γ-H2AX, suggesting that the integrity of the genomic DNA was indeed compromised (Figure 4a).

Based on the romidepsin-mediated inhibition of cell proliferation (Figure 1) and activation of DNA-damage response (Figure 4a), we investigated if activation of apoptosis was a contributory factor in the response to this drug. Increased cleavage of PARP1 and caspase 3 was observed in the cell lines exposed to at least 10 nm romidepsin (Figure 4a). The level of the pro-apoptotic protein BAK increased in SUPT1 but not in PEER cells, which may be related to their p53 status. The level of tumor suppressor protein p21^Waf1/Cip1^, an inhibitor of cyclin-dependent kinases, also increased and the level of the anti-apoptotic XIAP protein correspondingly decreased (Figure 4a). These results demonstrate that romidepsin-mediated damage to DNA and blockage of cell cycle progression correlate with the induction of apoptosis.

Again, the potential clinical relevance of these observations is shown by the similarly increased phosphorylation of histone 2AX and cleavage of PARP1, caspase 3 and caspase 9 in the T-cell lymphoma patient cell sample exposed to romidepsin (Figure 4b). Significant activation of apoptosis was observed at 5 nm romidepsin, suggesting even greater sensitivity of the patient cell sample to romidepsin compared with the established cell lines PEER and SUPT1.

### Romidepsin activates the SAPK/JNK stress signaling pathway

**Figure 5:**
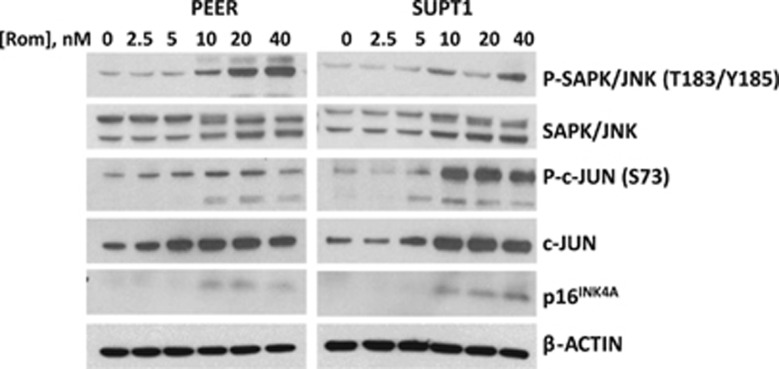
Exposure of malignant T cells to romidepsin (Rom) activates the SAPK/JNK stress signaling pathway. Cells were exposed to romidepsin for 48 h prior to western blot analysis.

The observed production of ROS and perturbation of mitochondria (Figure 3) suggest a role for romidepsin-mediated activation of stress pathways leading to apoptosis. We, therefore, sought to determine the effects of this drug on the activation of the stress-activated protein kinase/c-Jun N-terminal kinase (SAPK/JNK) signal transduction pathway, which is known to transmit and convert stress signaling into apoptosis signaling in various cell types.^20^ Increased phosphorylation of SAPK/JNK at threonine 183/tyrosine 185 was observed in PEER and SUPT1 cells exposed to at least 10 nm romidepsin. Analysis of its downstream target genes including *c-JUN* and *CDKN2A* (which encodes p16^INK4A^) showed upregulation of their expression as indicated by an increase in their protein level, and increased phosphorylation of c-JUN, at least in SUPT1 cells (Figure 5). As p16^INK4A^ protein is a cyclin-dependent kinase inhibitor that negatively regulates the cell cycle, our results suggest that romidepsin cytotoxicity is partly due to inhibition of cell cycle progression through activation of the SAPK/JNK pathway. This observation is consistent with increased p21^Waf1/Cip1^, another cyclin-dependent kinase inhibitor, in the presence of romidepsin, as described above (Figures 4a and b).

### Romidepsin increases the level of proteins involved in the UPR

**Figure 6:**
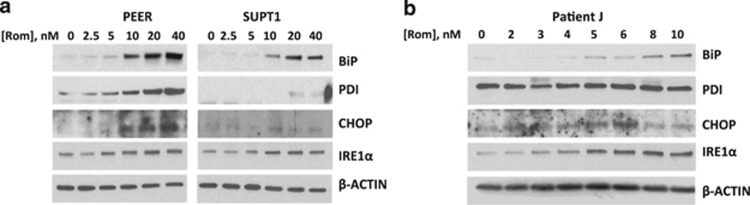
Western blot analysis of proteins involved in the unfolded protein response. Cells were exposed to romidepsin (Rom) for 48 h prior to analysis of total cell extracts from cell lines (**a**) and patient J cell sample (**b**).

In search of other mechanisms underlying the observed romidepsin-mediated apoptosis in malignant T cells, we examined the effects of this drug on the endoplasmic reticulum (ER), where secretory and transmembrane proteins are modified and properly folded. Molecular chaperones facilitate protein folding, and their upregulation is indicative of ER stress. Analysis of some of these chaperones in romidepsin-treated cells showed increased levels of the ER-binding protein BiP (Figure 6), suggesting activation of the unfolded protein response (UPR).^21^ Protein disulfide isomerase, another protein chaperone that catalyzes the formation and isomerization of disulfide bonds in the ER,^22^ also increased in PEER and SUPT1 cells exposed to romidepsin but not in patient J cells (Figure 6). Consistent with these findings is the observed increase in the level of C/EBP homologous protein, which triggers UPR and programmed cell death during ER stress.^23^ C/EBP homologous protein is a transcription factor associated with expression of apoptosis-related genes.^24^ The level of inositol-requiring enzyme 1α, a protein that possesses both kinase and endonuclease activities and is known to transmit the unfolded protein signal across the ER membrane,^25^ also increased in cell lines and in the patient cell sample after exposure to romidepsin (Figure 6). Taken together, these results suggest that romidepsin causes ER stress in malignant T cells, which may consequently trigger UPR and cell death.

### Romidepsin downregulates the phosphatidylinositol 3-kinase/AKT/mammalian target of rapamycin (PI3K-AKT-mTOR) pathway

**Figure 7:**
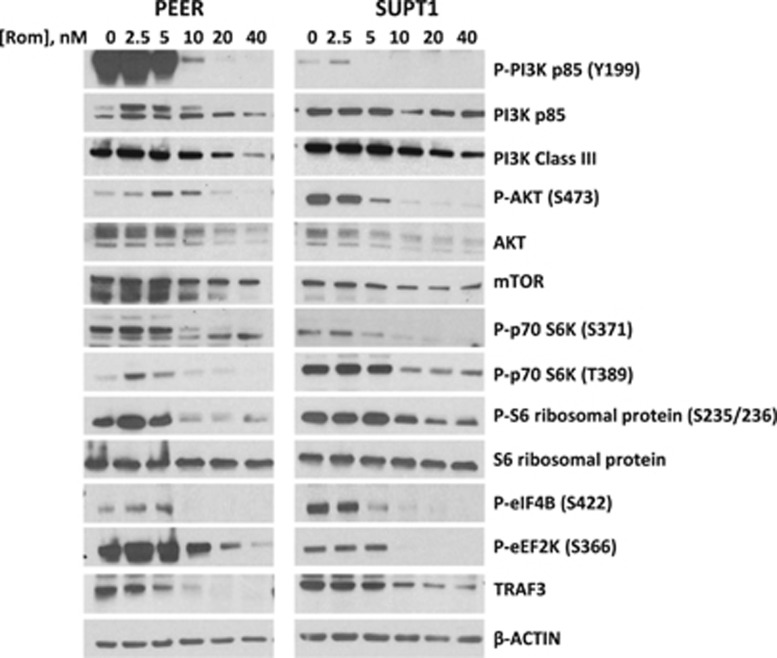
Western blot analysis of phosphatidylinositol 3-kinase (PI3K) and its downstream signaling components in the PI3K-AKT-mTOR pathway. Cells were exposed to romidepsin (Rom) for 48 h.

Romidepsin has been reported to inhibit PI3K in prostate and colorectal cancer cell lines.^8^ We wanted to know if the drug has similar effects in malignant T cells. Exposure of PEER and SUPT1 cells to at least 5 nm romidepsin decreased the phosphorylation of PI3K p85 (a regulatory subunit of PI3K) at tyrosine 199 (Figure 7). A similar decrease in the level of PI3K class III was also observed. AKT is downstream in the PI3K signaling pathway. Romidepsin decreased the level of AKT as well as its serine 473-phosphorylated form. The level of downstream mTOR was also decreased by romidepsin. These observations correlate with decreased phosphorylation of p70 S6 kinase, S6 ribosomal protein, eIF4B and eEF2K (Figure 7), which are all regulated by AKT-dependent mTOR and involved in protein synthesis. A recent report demonstrated that TRAF3 is important for continued PI3K/AKT signaling^26^ and our results show that romidepsin exposure decreased the level of TRAF3 in malignant T cells (Figure 7), suggesting extended suppression of the PI3K/AKT pathway and inhibition of protein translation, further compromising cell survival.

### Romidepsin-mediated inhibition of the Wnt/β-catenin pathway

**Figure 8:**
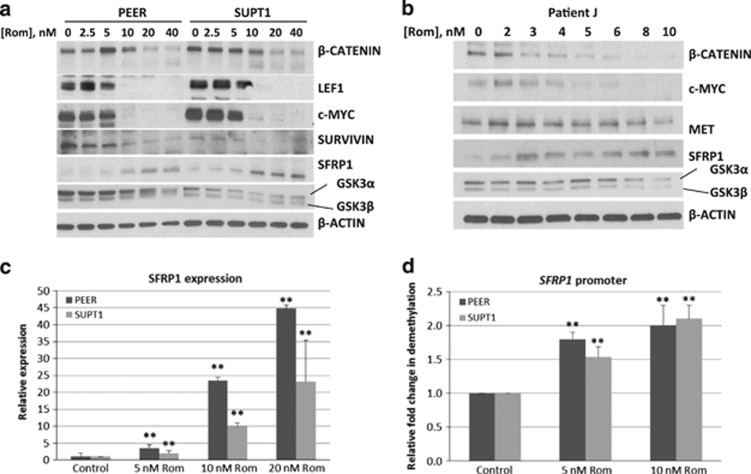
Romidepsin-mediated downregulation of the β-catenin pathway. Cells were exposed to the indicated concentrations of romidepsin (Rom) for 48 h. Total cell extracts were analyzed by western blot (**a** and **b**). Total RNA was used to prepare cDNA, which was analyzed for expression of SFRP1 by RT-PCR (**c**). Genomic DNA was analyzed for the extent of demethylation of the *SFRP1* gene promoter by methylation-specific PCR (**d**). The results (**c** and **d**) are averages of two independent experiments; each RT-PCR was done in four replicates. The double asterisks indicate a statistically significant difference (*P*<0.05) when compared with the corresponding control.

The Wnt/β-catenin survival pathway has been a target for drug discovery in the context of treatment of hematological malignancies due to its constitutive activation therein.^27^ We therefore examined the effects of romidepsin in malignant T cells. A dose-dependent decrease in the level of β-catenin was observed in PEER, SUPT1 and patient J samples (Figures 8a and b). The appearance of a cross-reacting lower molecular weight protein correlates with a decrease in the level of β-catenin in the two cell lines, suggesting possible protein cleavage.

Active β-catenin is known to translocate to the nucleus and interact with LEF1 to upregulate the expression of canonical target genes.^28^ Like β-catenin, the level of LEF1 protein decreased in cells exposed to romidepsin (Figure 8a). The decreased levels of these two transcription factors suggest possible downregulation of their target genes. In fact, the expression of selected target genes such as *c-MYC*, *SURVIVIN* and *MET*^29^ decreased as shown by a reduction in their protein levels in cells exposed to at least 10 nm romidepsin (in established cell lines) or 4 nm romidepsin (in patient J cell sample). Concomitantly, we observed an increase in the level of SFRP1 protein (Figures 8a and b); SFRP1 is a known antagonist of the Wnt/β-catenin pathway and its binding to Wnt triggers a cascade of events leading to β-catenin degradation.^30^ β-catenin is also negatively regulated by GSK3β.^31^ However, our results show a decrease in the level of GSK3α and GSK3β in the presence of romidepsin, suggesting that GSK3β may not be involved in the romidepsin-mediated downregulation of the β-catenin pathway in this experimental model.

DNA demethylation is known to increase expression of the *SFRP1* gene.^30^ To initially determine whether the regulation of SFRP1 expression is occurring at the transcription level, we used RT-PCR to measure changes in its mRNA levels in cells exposed to romidepsin. In both PEER and SUPT1 cells, a significant increase in the expression of SFRP1 mRNA was observed (Figure 8c). Its expression increased 10–22-fold in the presence of 10 nm romidepsin and further increased at higher drug concentrations. Analysis of the methylation status of the *SFRP1* gene promoter by methylation-specific RT-PCR showed an approximately twofold increase in its demethylated form (Figure 8d). Overall, these results suggest that romidepsin causes hypomethylation of the *SFRP1* gene promoter, increases its expression, downregulates β-catenin and inhibits expression of its downstream pro-survival target genes.

## Discussion

The Food and Drug Administration approval of romidepsin for treatment of cutaneous and peripheral T-cell lymphoma axiomatically reflects its efficacy against tumor T cells. However, the details of its mechanisms of cytotoxicity, and effects on T-cell lymphoblastic leukemia/lymphoma cell lines, remain unknown. Here, we provide *in vitro* evidence showing the activation of apoptosis and modulation of multiple survival pathways in established malignant T-cell lines and a primary T-cell lymphoma patient sample, which may underlie the efficacy of romidepsin. The observed apoptosis correlates with an increased DNA-damage response, ROS production and mitochondrial damage. The commitment of malignant T cells to cell death also correlates with activation of the SAPK/JNK stress signaling and UPR pathways, as well as inhibition of the PI3K-AKT-mTOR and Wnt/β-catenin pathways.

Romidepsin is known to inhibit HDAC but its effects on other enzymes remain to be identified. Increased acetylation of histones affects the integrity of the chromosome with concomitant changes in DNA replication, repair and transcription. Acetylation of histones causes replication fork delays, increasing DNA double-strand break formation.^19^ Such DNA damage is similarly suggested here by increased phosphorylation of histone 2AX in cells treated with romidepsin (Figure 4), which may be exacerbated by the observed increased ROS production in romidepsin-treated malignant T cells (Figure 3). This result is consistent with increased production of ROS by other HDAC inhibitors in solid tumors^5^ and in HL-60 leukemia cells.^4^ Such changes in the cellular redox status also compromise the integrity of the mitochondrial membrane. Exposure of malignant T cells to romidepsin decreases the MMP, which may cause leakage of pro-apoptotic factors from mitochondria to the cytoplasm and activation of caspases (Figures 3 and 4). This mitochondria-mediated activation of intrinsic apoptosis is further suggested by the cleavage of PARP1 (Figure 4).

DNA-damage response and ROS production put malignant T cells under biological stress as suggested by the upregulation of the SAPK/JNK pathway (Figure 5). A downstream target of this pathway is the cyclin-dependent kinase inhibitor p16^INK4A^, which may contribute to cell cycle arrest. The increased level of p16^INK4A^ (Figure 5) and p21^Waf1/Cip1^ (Figure 4) in malignant T cells exposed to romidepsin suggests drug-mediated inhibition of cell cycle and may contribute to the observed cytotoxicity (Figure 1).

Cellular stress may also compromise the integrity of the ER where secretory and transmembrane proteins are modified and properly folded. The accumulation of unfolded proteins transduces signals to the nucleus and triggers apoptosis. Activation of the UPR is suggested by the increased level of protein chaperones BiP and protein disulfide isomerase, the pro-apoptotic transcription factor C/EBP homologous protein, and the endoribonuclease/kinase inositol-requiring enzyme 1α in malignant T cells exposed to romidepsin (Figure 6). Whether this activation of UPR depends on the inhibition of deacetylases, ROS production and/or other unknown biological effects, remains to be elucidated.

An oncogenic pathway inhibited by romidepsin in malignant T cells is the PI3K-AKT-mTOR axis. One major target of PI3K is AKT, directly or through PDK1, which affects the translation machinery through mTOR. Romidepsin decreases the phosphorylation of the p85 regulatory subunit of PI3K, which correlates with the observed decrease in the phosphorylation status of AKT (Figure 7). As the level of pan AKT also decreases, it is possible that romidepsin affects both expression and phosphorylation of AKT. Downstream of AKT is mTOR, a kinase that phosphorylates p70 S6 kinase. Romidepsin exposure results in the inhibition of the phosphorylation of p70 S6 kinase that may invoke, through a cascade of events, inhibition of protein translation.

Another pro-survival target of romidepsin is β-catenin. When activated in the cytoplasm, β-catenin translocates to the nucleus, pairs with LEF1 and other transcription factors, and mediates transcription of pro-survival genes including *c-MYC* and *SURVIVIN*.^29^ We show in the present study that romidepsin decreases the level of β-catenin and LEF1 as well as their downstream targets c-MYC and SURVIVIN (Figure 8). β-catenin is negatively regulated by several proteins including GSK3β, SFRP1, DKK3 and WIF1.^30, 31^ SFRP1, but not GSK3β, increased with romidepsin exposure in a dose-dependent manner, suggesting a negative correlation between SFRP1 and β-catenin expression (Figure 8). The increase in SFRP1 expression occurs at both the transcription and translation levels (Figure 8). The observed transcriptional activation is most likely due to hypomethylation of the *SFRP1* gene promoter (Figure 8).

In addition to its downregulation, β-catenin was also found to be cleaved in malignant T cells exposed to romidepsin (Figure 8). Based on the previously reported apoptosis-associated cleavage of β-catenin by caspase 3 seen in other studies,^32^ this effect is probably mediated by caspase 3 activation in the sub-population of cells undergoing apoptosis.

The DNA hypomethylating activity of romidepsin has not been previously reported. Here, we show evidence that the drug decreases the level of DNA methylation of the *SFRP1* genes. Furthermore, the levels of DNA methyl transferases DNMT1 and DNMT3B also decrease in romidepsin-treated cells (Figure 2). How romidepsin causes DNA hypomethylation remains unknown. Whether it directly binds to DNA methyl transferases (as with 5-aza-2′-deoxycytidine) or inhibits S-adenylhomocysteine hydrolase (as with cladribine)^33^ requires further study.

In conclusion, our data are generally consistent with the reported biological effects of other HDAC inhibitors such as SAHA, panobinostat, entinostat and mocetinostat^34, 35^ as far as induction of apoptosis (owing to DNA-damage, ROS production, mitochondrial damage and inhibition of the pro-survival PI3K-AKT pathway) is concerned. We present new lines of evidence showing romidepsin cytotoxicity to T-cell lymphoblastic cells via multiple mechanisms including (a) upregulation of proteins involved in cell cycle arrest, (b) activation of the UPR and stress signaling pathways and (c) the associated DNA hypomethylating activity of romidepsin, which may invoke the inhibition of the oncogenic Wnt/β-catenin pathway. All of these affected pathways, together with the above-mentioned mitochondria-dependent cell death, converge to a common point that commit cells to apoptosis and may explain the efficacy of romidepsin in cutaneous T-cell lymphoma and other T-cell malignancies.
