# Supplementary material for: Romidepsin targets multiple survival signaling pathways in malignant T cells
Source: Blood Cancer J. 2015 Oct 16;5(10):e357–. doi: 10.1038/bcj.2015.83 (PMC4635192; doi:10.1038/bcj.2015.83)
Supplement: Supplementary Table [file bcj201583x1.docx]

Table 1. List of primary antibodies, their sources and dilutions

 Valdez *et al*. Supplementary Materials
